# Dynamicity and persistence of severe acute respiratory syndrome coronavirus-2 antibody response after double dose and the third dose with BBV-152 and AZD1222 vaccines: A prospective, longitudinal cohort study

**DOI:** 10.3389/fmicb.2022.942659

**Published:** 2022-08-09

**Authors:** Debaprasad Parai, Hari Ram Choudhary, Girish Chandra Dash, Susmita Behera, Narayan Mishra, Dipti Pattnaik, Sunil Kumar Raghav, Sanjeeb Kumar Mishra, Subrat Kumar Sahoo, Aparajita Swain, Ira Mohapatra, Matrujyoti Pattnaik, Aparnamayee Moharana, Sandhya Rani Jena, Ira Praharaj, Subhra Subhadra, Srikanta Kanungo, Debdutta Bhattacharya, Sanghamitra Pati

**Affiliations:** ^1^Department of Microbiology, ICMR-Regional Medical Research Centre, Bhubaneswar, Odisha, India; ^2^Maharaja Krushna Chandra Gajapati Medical College and Hospital, Brahmapur, Odisha, India; ^3^The Chest Clinic, Brahmapur, Odisha, India; ^4^Department of Microbiology, Kalinga Institute of Medical Sciences, Bhubaneswar, Odisha, India; ^5^Institute of Life Sciences, Bhubaneswar, Odisha, India; ^6^Department of Community Medicine, Veer Surendra Sai Institute of Medical Sciences and Research, Burla, Odisha, India

**Keywords:** SARS-CoV-2, spike glycoprotein, neutralizing antibody, healthcare worker, BBV-152 and AZD1222

## Abstract

**Introduction:**

Vaccines are available worldwide to combat coronavirus disease-19 (COVID-19). However, the long-term kinetics of the vaccine-induced antibodies against severe acute respiratory syndrome coronavirus-2 (SARS-CoV-2) have not been sufficiently evaluated. This study was performed to investigate the persistence and dynamicity of BBV-152 (Covaxin)- and AZD1222 (Covishield)-induced immunoglobulin-G (IgG) antibodies over the year and neutralizing antibodies’ status after 1-month of booster dose.

**Materials and methods:**

This 52-week longitudinal cohort study documented antibody persistence and neutralizing antibodies status among 304 healthcare workers (HCWs) from six hospitals and research facilities in Odisha, enrolled during January 2021 and continued till March 2022. IgG antibodies against spike receptor-binding domain (RBD) of SARS-CoV-2 were quantified in an automated chemiluminescence immune assay-based (CLIA) platform and a surrogate virus neutralization test (sVNT) was performed by enzyme-linked immunosorbent assay (ELISA).

**Results:**

Among these 304 HCWs vaccinated with double doses, 154 HCWs (50.66%) were Covaxin recipients and the remaining 150 (49.34%) were Covishield recipients. During the follow-ups for seven times, a total of 114 participants were identified as vaccine breakthrough cases. In 190 non-infected HCWs, the median antibody titer was significantly waned from DD2 to DD10, both for Covaxin (231.8 vs. 42.7 AU/ml) and Covishield (1,884.6 vs. 369.2 AU/ml). No statistically significant differences in antibody titers were observed based on age, gender, comorbidities, and blood groups. The median inhibition activity of sVNT increased from 23.8 to 91.3% for Covaxin booster recipients and from 41.2 to 96.0% for Covishield booster recipients. Among 146 booster dose recipients, 48 were breakthrough cases after booster and all were contracted by the omicron variant.

**Conclusion:**

This year-long follow-up study found a 7- and 5-fold antibody waning in Covaxin and Covishield recipients, respectively, without any breakthrough infection history. However, individuals with booster breakthrough had mild symptoms and did not require hospital admission. The data also indicate the possible escape of omicron variants despite the presence of vaccine-induced neutralizing antibodies.

## Introduction

Vaccines against severe acute respiratory syndrome coronavirus-2 (SARS-CoV-2) have been rolled out worldwide to curb the severity of the coronavirus disease-19 (COVID-19). Despite the unprecedented efforts to contain COVID-19, the number of cases worldwide rose to 500 million and the pandemic cost more than 6 million lives by the end of April 2022 (World Health Organization, 2022b). The United States of America, India, and Brazil are the three leading countries of the COVID-19 cases worldwide, wherein India reported almost 43 million total cases with 0.52 million deaths by 2nd May 2022 (World Health Organization, 2022b). One of the primary reasons behind this increased number of cases is the continuous evolution of newer variants of SARS-CoV-2, making it more challenging in diagnosis, treatment, and vaccine development (Fernandes et al., 2022). The World Health Organization (WHO) approved various vaccines against COVID-19 at different phases, which include inactivated whole virion-based (CoronaVac by Sinovac, BBIBP-CorV or Covilo, and BBV152 or Covaxin) messenger RNA (mRNA)-based, mRNA-based (mRNA-1273 by Moderna and BNT162b2 by Pfizer-BioNTech), vector-based (Ad26.COV2.S by Johnson & Johnson and ChAdOx1 nCoV-19/AZD1222 or Covishield), and protein subunit-based (NVX-CoV2373 by Novavax) vaccines (World Health Organization, 2022a).

India initiated the world’s largest vaccination campaign against COVID-19 on 16 January 2021 when the Indian Government and Drugs Controller General of India (DCGI) gave the emergency approval of BBV-152 (Covaxin) and AZD1222 (Covishield) as the two preliminary vaccines. Covaxin is made indigenously by Bharat Biotech, India, using an inactivated whole-virion vero cell against SARS-CoV-2. Covaxin was found to have 77.8% efficacy against symptomatic and 63.6% protection after 14 days of two doses against asymptomatic COVID-19 (Ella et al., 2021). Covishield is a recombinant, vector-based vaccine against SARS-CoV-2 spike protein produced indigenously by Serum Institute, India. The overall efficacy of this vaccine was 74.0% and a maximum of 100% in severe or critical symptomatic COVID-19 at ≥15 days after the second dose (Falsey et al., 2021). Till 9th May 2022, 1.53 billion doses of Covishield have been administered in the country vs. 318 million shots of Covaxin (Ministry of Health and Family Welfare, 2021).

The omicron variant first detected in Southern Africa in November 2021 was highly transmissible and led to another pandemic wave that hit the world, including India. The BA.1.1 and BA.2 were the most predominant lineages among the 21 different sub-lineages found across 164 countries, causing many vaccine breakthrough infections (Bansal and Kumar, 2022; Chen et al., 2022; Mohapatra et al., 2022; Yadav et al., 2022). A third dose of the vaccine was initiated by the end of 2021 in many countries worldwide to manage the waning immunity and the immune escape by the newly emerged SARS-CoV-2 variants (Tartof et al., 2021; Wu et al., 2022). The Government of India has initiated the homologous precautionary or booster dose shot for healthcare workers (HCWs), frontline workers, and those above 60 years or who have comorbidities from 10 January 2022. India has already administered vaccines to 29 million individuals till the first week of May 2022 (Ministry of Health and Family Welfare, 2021).

This study investigated the year-long longitudinal anti-S receptor-binding domain (RBD) IgG responses and their dynamicity in Covaxin and Covishield vaccine recipients. Both the vaccines effectively induced humoral responses, which tend to decline over the year. Efficacy of the third dose was also measured by evaluating the neutralizing antibodies and anti-S RBD IgG. Diminished neutralization potency against omicron variants was observed among both the vaccine recipients indicating the need for more robust vaccine options.

## Materials and methods

### Study settings

The enrollment for this year-long longitudinal cohort study started in January 2021 and continued till March 2022. A total of 304 HCWs from six different hospitals, healthcare facilities, and research institutes in Odisha, India, were included in this cohort study. All the samples were sent maintaining a proper cold chain and tested at the Cobas Laboratory of Indian Council of Medical Research-Regional Medical Research Centre (ICMR-RMRC), Bhubaneswar, India.

### Study design

Three milliliters of blood samples were collected from all the participants at different timepoints to analyze neutralizing antibodies against SARS-CoV-2 and IgG against the spike RBD of SARS-CoV-2. The first sample was collected at baseline (before a dose of vaccine) and subsequently at 1 month of single dose (SD1), 1 month (DD1), 2 months (DD2), 3 months (DD3), 5 months (DD5), and 10 months (DD10) after double dose of vaccine. We also evaluated a subset of 146 participants among the 304 HCWs who were eligible for a subsequent booster dose. Blood samples were collected at baseline (before a third dose of vaccine; BD0) and after 1 month of booster dose (BD1) from those participants. Participants who developed symptoms suggestive of COVID-19 were instructed to send their nasopharyngeal swab (NPS) sample at ICMR-RMRC, Bhubaneswar, for the confirmation of COVID-19 using real-time reverse-transcriptase polymerase-chain-reaction (RT-PCR). In addition, samples from the participants diagnosed with COVID-19 during routine RT-PCR tests were also sent by their respective institutions to the Cobas laboratory at Bhubaneswar for further confirmation.

### Participant enrollment and data collection

Study participants were recruited voluntarily during 15th December 2020 to 15th January 2021 after a notification was published in all the participating institutions. Initially, a total of 614 HCWs were recruited in the cohort following the study inclusion criteria and were followed initially for 6 months after being reported. Post initial follow-up and after dropout, we ended up with 304 eligible participants who consented to be followed up for another 6 months. All were explained about the prospect of the study and written informed consent was obtained from each, prior to the enrollment. The inclusion criteria were as follows: (i) participants should be ≥18 years old; (ii) had no COVID-19 history in the last 6 months; (iii) willing to take either Covaxin or Covishield vaccine against SARS-CoV-2; (iv) ready to give blood samples; (v) ready to provide study-related personal information; and (vi) signed the written informed consent. All the 146 participants who took only the same vaccine type during booster were further followed for a month. The relevant demographic data were collected from all participants at the baseline and SARS-CoV-2 infection status was updated at each mentioned timepoint during the blood sample collection.

### Ethical approval

The study was approved by the Institutional Human Ethical Committee of ICMR-Regional Medical Research Centre, Bhubaneswar (ICMR-RMRCB/IHEC-2020/036 dated 07/11/2020).

### Severe acute respiratory syndrome coronavirus-2 antibody test method

Serum was separated from the collected blood samples and subjected to antibody tests. IgG titer against spike RBD of SARS-CoV-2 was quantified by chemiluminescent microparticle immunoassay (CMIA)-based ARCHITECT i1000SR platform from Abbott Diagnostics (Chicago, IL, United States) using manufacturer-provided ARCH SARS-CoV-2 IgG II Quant kit. A cutoff value of ≥50 AU/ml was considered as positive. An enzyme-linked immunosorbent assay (ELISA)-based surrogate virus neutralization test (sVNT) was performed by a commercially available kit (from GenScript Biotech, New Jersey, NJ, United States) to detect circulating neutralizing antibodies against SARS-CoV-2 that block the interaction between the RBD with angiotensin-converting enzyme 2 (ACE2), a cell surface receptor. A minimum inhibitory percentage (%I) of 30% was considered as the presence of neutralizing antibodies.

### Confirmation of severe acute respiratory syndrome coronavirus-2 by reverse-transcriptase polymerase-chain-reaction and variant determination by next-generation sequencing

The NPS samples were collected from the booster dose recipients who presented with COVID-19 symptoms after a minimum of 15 days from the date of booster administration. Samples were further processed in an automated nucleic acid extractor Maelstrom 4800 (Taiwan Advanced Nanotech Inc., Taiwan, China) for RNA extraction. RT-PCR was set using a commercial kit OmiSure (TATA Medical and Diagnostics, Chennai, India), which was intended to detect SARS-CoV-2 Omicron variants. Data were analyzed as per the guidelines given by the Indian Council of Medical Research (ICMR), New Delhi.

A subset of 20% (*n* = 10) of omicron-positive samples were further sent for next-generation sequencing (NGS) to estimate the most predominant sub-lineage of circulating omicron variants. NGS was performed in the Oxford Nanopore MinION Mk1C (Oxford Nanopore Technologies, Oxford, United Kingdom) sequencing platform using “Midnight protocol” primer set (Version: PCTR_9125_v110_revE_24Mar2021) (Pembaur et al., 2021). Briefly, extracted RNA was converted to complementary DNA (cDNA) using LunaScript™ RT SuperMix (New England BioLabs, MA, United States) followed by a multiplex PCR, which generates consecutively tiled, non-overlapping 1,200 bp amplicons in two sets to avoid overlaps but to cover the entire SARS-CoV-2 genome. Rapid barcodes were added to each sample using the rapid barcoding sequencing kit (SQK-RBK-110.96; Oxford Nanopore Technologies, Oxford, United Kingdom) following the manufacturer’s recommendations. Then, the samples were pooled and subjected to clean-up using solid-phase reversible immobilization (SPRI) beads. Sequencing was performed with the options “basecalling” and “demultiplexing” being enabled, both performed by the in-built “guppy” algorithm (version 5.0.1). Sequencing was stopped after reaching at least 10 Mb for each barcode. After filtering the processed reads using the field bioinformatics pipeline, reads were aligned to the reference genome (MN908947.3). Variant calling was done using Medaka workflow from reads that were aligned. Consensus FASTA was generated with Samtools with the latest version of SnpEff used for annotation of variants with NC_045512. For lineage classification, the Phylogenetic Assignment of Named Global Outbreak Lineages (Pangolin) software suite was used (Rambaut et al., 2020). ARTIC field bioinformatics and Nextclade were the other two pipelines used for bioinformatic analysis (Loman et al., 2020; Aksamentov et al., 2021). Sequences were deposited and accession numbers were obtained.

### Statistical analysis

Descriptive statistical analyses were performed by the SPSS software (IBM SPSS Statistics for Windows, version 24.0, Armonk, NY, United States) and GraphPad Prism 9.3.1 for Windows (GraphPad Software, La Jolla, CA, United States). The statistical significance was set at *p* < 0.05.

## Results

**FIGURE 1:**
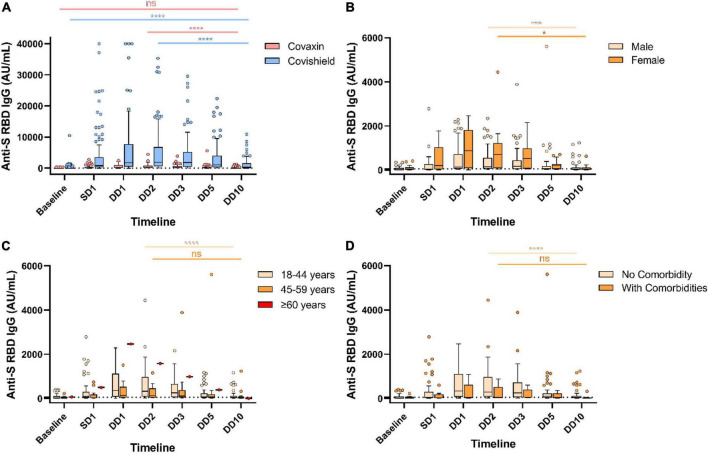
Decreased humoral immune response against severe acute respiratory syndrome coronavirus-2 (SARS-CoV-2) vaccines over the year. Anti-spike RBD IgG antibody responses at different timepoints in Covaxin and Covishield double dose recipients without any breakthrough infection **(A)**. Anti-S RBD IgG antibody levels of Covaxin recipients stratified by gender **(B)**, age **(C)**, and comorbidities **(D)**. ns, non-significant; *p < 0.05; ***p < 0.001; ****p < 0.0001. Tukey method was used to plot whiskers. SD1, 1 month after single dose; DD1, 1 month; DD2, 2 months; DD3, 3 months; DD5, 5 months; DD10, 10 months after double dose.

Among 304 HCWs, 209 (68.8%) were men, and the remaining 95 (31.2%) participants were women. The median age of the participants was calculated at 39 years with an interquartile range (IQR) of 29–48 years. A total of 154 (50.7%) individuals took Covaxin and another 150 (49.3%) received Covishield vaccine. In the Covaxin recipient group, 114 were male and 40 were female participants with a cumulative median age of 35 years (IQR: 28–44). Details of the participants are given as a Metadata 1 in Supplementary Material. The seroconversion rate after 1 month of complete dose was 68.3% in the Covaxin group vs. 95.8% in the Covishield group. At baseline, the median titer of Covaxin recipients was 6.7 AU/ml (IQR: 2.6–64.9). The numbers of men and women were 95 and 55, respectively, among Covishield receivers with a median age of 44 years (IQR: 33–51). The median titer at baseline was calculated at 55.7 AU/ml (IQR: 3.1–117.4) for this vaccine group (Figure 1A).

**FIGURE 2:**
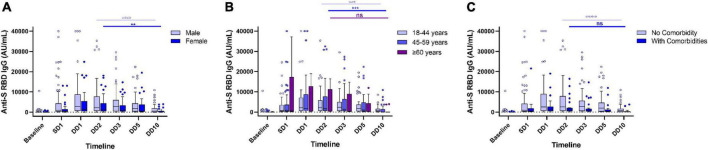
Waning of humoral immune response in Covishield recipients without any breakthrough infection. Levels of anti-spike RBD IgG antibody stratified by gender **(A)**, age **(B)**, and comorbidities **(C)** in Covishield recipients at different timepoints during a year-long follow-up. ns, non-significant; **p < 0.01; ***p < 0.001; ****p < 0.0001. Tukey method was used to plot whiskers. SD1, 1 month after single dose. DD1, 1 month; DD2, 2 months; DD3, 3 months; DD5, 5 months; DD10, 10 months after double dose.

Our data presented significant differences in antibody responses among the two vaccines. The antibody titer of 190 HCWs (out of 304) without any post-vaccination COVID-19 history showed a significant waning at the end of 10 months from the double dose of vaccination. Maximum antibody titer was noted after 2 months of complete vaccination (at DD2) for both Covaxin and Covishield recipients. The level of anti-spike (S) RBD IgG was significantly (*p* < 0.0001) higher among Covishield recipients when compared with Covaxin recipients at SD1 (Table 1). The highest Covaxin-induced antibody concentration was recorded as 231.8 AU/ml (IQR: 71.6–820.9) at DD2, which started to wane from DD3 (median = 209.2 AU/ml; IQR: 57.8–604.8) and reached 42.7 AU/ml (IQR: 6.5–110.5) after a 7-fold decrease at the end of 10 months (DD10) post-vaccination (Figure 1A). The level of IgG at DD10 among Covaxin recipients was found to be statistically similar to their baseline (8.75 AU/ml; IQR: 3.4–98.9). A total of 41 (53.9%) individuals among them were detected as seronegative with a median of 17.2 AU/ml (IQR: 2.4–32.6) at DD10. Among the Covishield recipients, the highest IgG concentration was recorded as 1,884.5 AU/ml (IQR: 573.2–6,779.0), which gradually decreased with time and was recorded as 369.2 AU/ml (IQR: 37.65–1,587.45) after a 5-fold drop at DD10 (Figure 1A). The median titer was 20.4 AU/ml (IQR: 5.7–36.6) among 34 (29.8%) participants who became seronegative at DD10. No statistical significance was observed in vaccine-induced antibody development across gender, age, blood groups, and comorbidities (Table 1 and Figures 1B, 2A,C). However, the persistence of IgG was higher, and the difference was statistically significant in the 45–59 year age group as compared to 18–44 year age group of Covaxin recipients (Figure 1C). In Covishield receivers, waning of antibody was considered non-significant (*p* = 0.166) among the ≥60 years age group (Figure 2B). Covaxin-induced IgG dropped faster among individuals with some comorbidities (hypertension, diabetes, any chronic disease, and ischemic heart disease) than in non-comorbid individuals as found in our study (Figure 1D).

**TABLE 1:** Demographics and baseline characteristics of cohort participants.

Infection status	Variables		*N* (%)	Baseline		SD1		DD1		DD2		DD3		DD5		DD10	
				Median (Q1–Q3)	*P*-value	Median (Q1–Q3)	*P*-value	Median (Q1–Q3)	*P*-value	Median (Q1–Q3)	*P*-value	Median (Q1–Q3)	*P*-value	Median (Q1–Q3)	*P*-value	Median (Q1–Q3)	*P*-value
**Non-Infected (*n* = 190)**	**Gender**	Male	129 (67.9)	24.3 (3.7–118.7)	0.31	278.1 (56.5–1,706.5)	0.75	786 (143.0–4,090.1)	0.87	665.4 (145.7–4,685.1)	0.78	547.1 (124.2–3,529.6)	0.78	264 (69.1–2,270.5)	0.58	81.6 (24.2–928.4)	0.38
		Female	61 (32.1)	16.1 (2.7–96.7)		473.9 (97.2–1,309.7)		1,082.9 (260.2–2,291.6)		873.5 (307.5–2,156.1)		773.5 (225.1–2,082.8)		277.18 (113.0–1,221.2)		83.1 (23.1–407.2)	
	**Age groups (years)**	18–44	115 (60.5)	24.1 (3.5–121.6)	0.86	278.1 (45.9–1,228.1)	0.10	766.8 (123.7–2,189.4)	0.19	663.12 (154.3–2,341.5)	0.38	561.9 (124.2–2,205.5)	0.35	233.7 (66.5–1,533.5)	0.12	105.2 (31.6–686.3)	0.20
		45–59	63 (33.2)	10.9 (3.5–99.1)		549.7 (136.5–2,279.3)		1,222.4 (224.6–5,449.3)		1,009.3 (210.5–4,850.5)		773.5 (189.2–3,883.2)		366.1 (116.7–3,761.9)		83.1 (23.5–967.5)	
		≥60	12 (6.3)	35.7 (2.4–130.8)		575.65 (162.0–13,240.5)		1,479.75 (290.9–12,579.2)		1,072.2 (292.6–10,781.1)		691.45 (212.4–8,688.3)		357.5 (133.1–4,164.4)		28.5 (3.7–88.6)	
	**Blood groups**	A	45 (23.7)	24.1 (3.8–133.3)	0.64	278.3 (109.8–858.4)	0.94	933.4 (129.7–2,400.1)	0.96	718.6 (167.3–2,171.8)	0.99	562.4 (203.2–3,186.2)	0.98	333.7 (94.6–2,637.9)	0.93	62.4 (14.6–758.4)	0.90
		B	55 (28.9)	18.4 (2–93.2)		391.5 (108.4–1,961.4)		725.5 (169.8–5,080.5)		590.8 (261.1–6,919.1)		501.8 (195.3–5,087.4)		270.6 (80.8–2,304.0)		80.9 (26.3–1,663.1)	
		AB	16 (8.4)	54.6 (5.1–120.8)		478.1 (37.2–1,699.6)		974.6 (124.5–7,545.2)		1,078.0 (154.3–4,906.7)		873.4 (144.3–3,426.6)		246.0 (49.1–2,912.1)		75.2 (23.5–724.4)	
		O	74 (38.9)	10.5 (2.7–101.6)		295.1 (112.7–1,569.5)		942.9 (255.7–2,270.1)		878.5 (237.3–2,170.6)		642.3 (219.2–2,168.9)		293.05 (107.1–1,572.4)		91.4 (29.5–805.9)	
	**Comorbidity**	Yes	34 (17.9)	7.0 (2.6–57.8)	0.04*	400.5 (107.7–1,621.7)	0.90	861.3 (280.5–1,963.3)	0.52	663.3 (234.7–1,537.1)	0.32	528.2 (183.2–1,174.9)	0.19	293.1 (85.8–578.9)	0.34	61.1 (4.9–208.2)	0.02*
		No	156 (82.1)	49.2 (3.8–121.4)		313.9 (53.9–1,529.7)		905.1 (142.3–4,249.6)		836.3 (187.6–4,537.4)		713.7 (187.7–3,368.1)		275.3 (76.8–2,634.9)		103.1 (31.7–947.9)	
	**Vaccine**	Covaxin	76 (40.0)	8.8 (3.4–98.9)	0.18	49.2 (17.2–282.9)	<0.0001*	179.9 (73.6–972.2)	<0.0001*	231.8 (71.6–820.9)	<0.0001*	209.2 (57.8–604.8)	<0.0001*	78.9 (32.7–227.5)	<0.0001*	42.7 (6.4–110.4)	<0.0001*
		Covishield	114 (60.0)	36.8 (3.4–121.7)		801.9 (250.1–3,526.4)		1,767.2 (504.7–7,704.0)		1,884.6 (573.2–6,779.0)		1,831.6 (441.5–5,201.6)		1,110.1 (244.7–4,034.0)		369.2 (37.6–1,587.4)	
**Infected (*n* = 114)**	**Gender**	Male	80 (70.2)	6.5 (2.4–60.7)	0.29	39.8 (18.9–264.6)	0.05	200.1 (74.0–753.3)	0.16	276.4 (91.1–667.3)	0.17	365.7 (105.1–1,573.5)	0.14	319.3 (79.9–6,832.6)	0.20	5,233.6 (1,805.8–13,425.8)	0.17
		Female	34 (29.8)	8.25 (2.4–75.6)		135.4 (25.9–767.7)		510.9 (60.5–1,511.4)		444.9 (86.8–1,508.5)		864.6 (156.3–13,049.8)		935.5 (78.6–18,359.3)		3,423.5 (1,113.9–8,824.4)	
	**Age groups (years)**	18–44	80 (70.2)	6.7 (2.6–61.5)	0.57	45.65 (24.1–345.2)	0.79	250.3 (74.0–1,096.1)	0.60	335.8 (83.1–969.5)	0.51	389.05 (120.2–4,405.4)	0.42	899.2 (81.8–8,497.5)	0.05	3,831.3 (1,373.6–12,622.1)	0.44
		45–59	32 (28.1)	6.6 (1.7–74.5)		91.5 (16.6–369.4)		223.7 (48.3–771.4)		235.1 (91.6–666.6)		413.3 (89.0–1,573.5)		214.9 (53.6–7,242.2)		5,277.7 (1,907.5–13,928.9)	
		≥60	2 (1.8)	391 (–)		1,086.9 (–)		2,013.1 (–)		1,391.8 (–)		20,222.8 (–)		32,213.7 (–)		8,792.7 (–)	
	**Blood groups**	A	23 (20.2)	2.7 (2.1–61.7)	0.53	32.2 (16.7–927.8)	0.84	574.1 (58.4–778.1)	0.34	357.1 (137.5–1,104.5)	0.49	448.9 (187.5–26,763.0)	0.58	3,315.3 (67.5–18,029.0)	0.59	3,265.4 (1,172.9–10,180.3)	0.06
		B	39 (34.2)	7.8 (3.7–76.8)		48.1 (15.8–335.6)		261.7 (53.7–751.4)		538.7 (113.4–1,007.3)		413.2 (79.51–789.1)		211.5 (41.9–914.1)		1,562 (1,025.9–7,076.3)	
		AB	7 (6.1)	7.2 (3.0–71.4)		45.7 (25.2–374.3)		322.6 (99.6–1,278.0)		373.4 (122.7–856.3)		413.5 (201.8–1,204.1)		489.5 (119.2–5,645.1)		2,937.3 (1,520.1–12,911.9)	
		O	45 (39.5)	7.1 (2.4–60.6)		53.6 (17.7–240.4)		149.5 (53.4–608.5)		137.2 (77.6–664.8)		345.7 (81.3–6,163.5)		555.4 (58.2–8,925.7)		8,306.3 (2,666.3–18,460.9)	
	**Comorbidity**	Yes	12 (10.5)	2.4 (1.7–59.6)	0.09	22.2 (5.6–320.6)	0.14	211.4 (27.7–744.6)	0.39	262.9 (68.4–550.3)	0.43	279.3 (86.8–1,113.1)	0.28	270.3 (49.9–1,0284.7)	0.65	2,779.1 (1,169.5–11,474.9)	0.50
		No	102 (89.5)	7.2 (2.5–64.2)		50.9 (24.8–362.2)		240.8 (77.2–1,089.6)		323.2 (88.6–892.7)		444.6 (118.9–4,093.4)		688.2 (85.0–8,792.9)		4,915.8 (1,676.0–13,083.2)	
	**Vaccine**	Covaxin	78 (68.4)	5.7 (2.3–10.3)	0.01*	27.7 (14.1–77.2)	<0.0001*	123.5 (45.4–529.0)	<0.0001*	137.5 (56.2–543.6)	<0.0001*	265.9 (79.3—806.2)	<0.0001*	204.7 (49.4–3,210.9)	<0.0001*	3,831.3 (1,124.1–11,496.7)	0.108
		Covishield	36 (31.6)	60.5 (2.6–113.2)		547.9 (236.8–1,149.9)		766.0 (294.6–2,009.8)		710.8 (351.1–2,376.8)		2,258.0 (510.0–20,360.3)		8,334.8 (2,579.8–24,372.5)		7,663.5 (1,818.0–13,928.9)	

*Significant at p-value <0.05.

**FIGURE 3:**
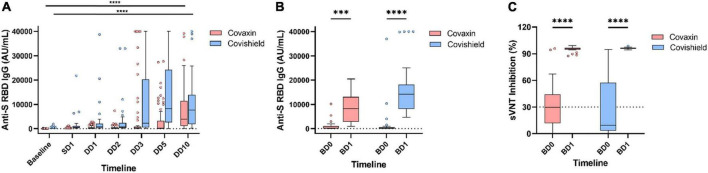
Longitudinal dynamics of anti-spike RBD IgG titer in coronavirus disease-19 (COVID-19) breakthrough infection. Antibody levels at different timepoints in participants who had COVID-19 after receiving double dose of Covaxin and Covishield **(A)**. Anti-spike RBD IgG **(B)** and neutralizing antibody **(C)** response in breakthrough cases after administration of booster. ^***^p < 0.001; ^*⁣*⁣**^p < 0.0001. Tukey method was used to plot whiskers. SD1, 1 month after single dose; DD1, 1 month; DD2, 2 months; DD3, 3 months; DD5, 5 months; DD10, 10 months after double dose; BD0, before booster dose; BD1, 1 month after booster dose.

**FIGURE 4:**
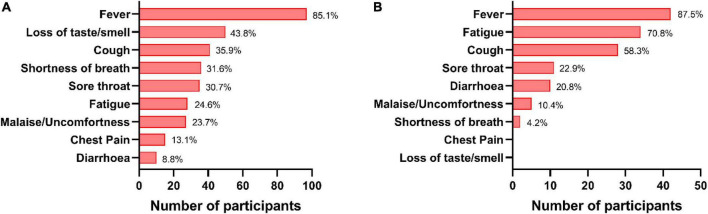
Status of symptoms in coronavirus disease-19 (COVID-19) cases. The percentages of various symptoms reported by the breakthrough cases post double dose vaccination **(A)** and post booster dose **(B)**.

During this year-long follow-up, 114 (37.5%) HCWs were infected by SARS-CoV-2 after double dose of either vaccine where 78 (68.4%) were Covaxin and 36 (31.6%) were Covishield recipients. The median day of these breakthrough cases was calculated as 77 days post double dose vaccination. The median anti-S RBD IgG level was quantified as 323.2 AU/ml (IQR: 37.3–2,123.9) at DD2, which was increased to 4,802.2 AU/ml (IQR: 665.8–22,413.7) among these breakthrough cases. However, the median antibody concentration was estimated as 240.8 AU/ml (IQR: 33.6–2,091.9) in participants immediately prior to breakthrough infection. The highest IgG median was observed at DD10 (3,831.3 AU/ml; IQR: 1,124.0–11,496.7) for Covaxin vs. DD5 (8,334.8 AU/ml; IQR: 2,579.9–24,372.6) for Covishield (Figure 3A). Among the vaccine breakthrough cases, 102 HCWs had mild symptoms, 7 individuals were hospitalized, none required ventilation, and no death was recorded. The primary symptom was fever among 97 (85.1%) HCWs and the other significant symptoms were as follows: loss of taste/smell (43.8%), cough (35.9%), shortness of breath (31.6%), sore throat (30.7%), fatigue (24.6%), and malaise (23.7%) (Figure 4A).

**FIGURE 5:**
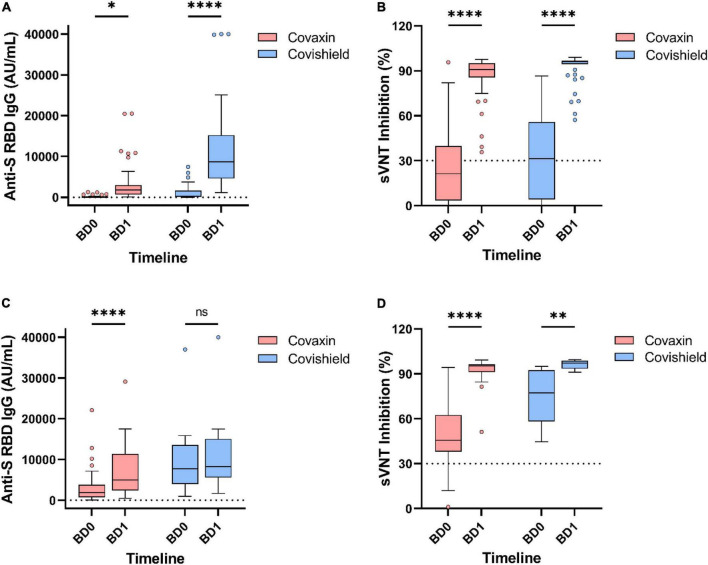
Antibody responses by different vaccines after 1 month of booster dose. Level of anti-spike RBD IgG antibody and percentage inhibition of neutralizing antibodies in booster recipients without any prior breakthrough infection **(A,B)** and with prior breakthrough infection **(C,D)**. ns, non-significant; *p < 0.05; ^**^p < 0.01; ^****^p < 0.0001. Tukey method was used to plot whiskers. BD0, before booster dose; BD1, 1 month after booster dose.

Among the 146 booster recipients, 94 had no history of vaccine breakthrough and were analyzed separately. The median IgG titer value among those Covaxin booster recipients was 42.9 AU/ml (IQR: 0.84–619.9) at BD0 and it was increased to 1,804.0 AU/ml (IQR: 233.7–10,606.7) after 1 month of booster administration. For Covishield booster recipients, the median titer of anti-S RBD IgG hiked from 229.9 AU/ml (IQR: 16.3–3,260.5) to 8,661.0 AU/ml (IQR: 2,335.1–22,698.8) at BD1. The inhibitory percentage of neutralizing antibodies was recorded at 90.95% and 96.0% among the Covaxin and Covishield booster dose recipients, respectively (Figure 5).

Booster recipients having breakthrough infection (*n* = 52) history prior to booster dose had a median IgG level of 2,156.4 AU/ml (IQR: 816.6–5,277.7) at BD0, which further raised to 6,123.7 AU/ml (IQR: 2,734.8–11,894.2) at BD1. The percentage of inhibition by neutralizing antibodies increased from 52.2% (IQR: 40.5–70.7) to 95.5% (IQR: 91.2–97.2) in those booster recipients regardless of the vaccine type (Figures 5C,D).

A total of 48 (32.9%) booster recipients were infected with SARS-CoV-2 at least 15 days after the booster dose and 13 (8.9%) of them were categorized as reinfected as per the definition (Mukherjee et al., 2021). Of these 48 recipients, 23 (47.9%) were administered Covaxin and the rest 25 (52.1%) were administered Covishield. Most of them (42; 87.5%) had only fever as the primary symptom followed by fatigue (34; 70.8%) and cough (28; 58.3%) (Figure 4B). None of them required any hospital admission. The median IgG antibody level was noted as 215.9 AU/ml (IQR: 3.5–4,469.1) at BD0 among the Covaxin booster dose beneficiaries, and the same was 76.3 AU/ml (10.8–6,551.5) as found in the Covishield group. Inhibitory percentage of neutralizing antibody among booster breakthrough was recorded at 29.7% (4.9–83.5) and 9.4% (0.0–93.4) for Covaxin and Covishield recipients, respectively (Figures 3B,C) at BD0. Among those 48 booster breakthrough cases, all were infected with the omicron variants of SARS-CoV-2 as found in the RT-PCR omicron detection test kit. The genome sequencing analysis further confirmed and identified the sub-lineages of those 10 omicron-positive samples as BA.2. The sequences were submitted to the public repository “Global Initiative on Sharing All Influenza Data” (GISAID) through the Indian SARS-CoV-2 Genomics Consortium (INSACOG) consortium after passing the GISAID quality control requirements. The accession numbers for the submitted sequences were EPI_ISL_12363379, EPI_ISL_12363383, EPI_ISL_12363394, EPI_ISL_12363395, EPI_ISL_12363399, EPI_ISL_12363385, and EPI_ISL_12363393. The details of the read length and genomic coverages of the sequenced data are given in a Supplementary Table 1.

## Discussion

The COVID-19 severity and the number of hospitalizations have been significantly reduced after the successful development of vaccines against SARS-CoV-2. However, most research articles have shown the short-term protection governed by those vaccines (Pritchard et al., 2021; Vasileiou et al., 2021; Andrews et al., 2022). Therefore, the current challenges include the status of long-term protection conferred by vaccine-induced antibodies and the need for booster doses. Our study found that a higher titer of IgG was developed by Covishield throughout all the timepoints compared with Covaxin, which was similar to the earlier short-term findings (Choudhary et al., 2021; Singh et al., 2021). The inhibitory percentage of neutralizing antibodies was still positive (31.4%) compared with being negative (21.2%) at the end of 10 months after complete vaccination (double dose). In this prospective longitudinal study, we found that a significant waning of humoral responses started as early as 3 months post double dose. The waning in anti-spike RBD IgG antibody titers and sero-reversion was the most prominent for Covaxin, which also had the lower IgG titers throughout the follow-ups among the participants having no history of natural infection. We also observed an overall higher antibody titer in female and non-comorbid participants, which can be corroborated with the data from earlier studies with different sets of vaccines (Levin et al., 2021; Barin et al., 2022; Sauré et al., 2022). The rapid waning of antibodies was noted among the older (≥60 years) age and comorbid groups in both the vaccine recipients. A total of 75 (39.4%) HCWs were converted to seronegative at DD10 among whom 41 (54.7%) were from the Covaxin arm vs. 34 (45.3%) from the Covishield arm. Antibody level in Covaxin recipients became non-significant (*p* = 0.211) after 10 months of complete dose, which clearly speculates the need for a booster dose at this timepoint.

The administration of homologous booster dose had increased both the anti-S IgG and neutralizing antibodies after 1 month of administration. Covaxin can boost around 25-fold (1,310.2 AU/ml; IQR: 378.2–2,327.5) in IgG titer level, whereas the Covishield booster spiked 3-fold (4,985.3 AU/ml; IQR: 2,697.1–8,997.1) in participants who never had COVID-19 history. Both the vaccine boosters were able to develop neutralizing antibodies at a 92% inhibitory level. We found a negative neutralizing antibody percentage at BD0 in participants who got infected with SARS-CoV-2 after a booster dose. However, the level of neutralizing antibodies was 56.2 and 58.6% for Covaxin and Covishield, respectively, at the time of infection after the booster dose. In booster breakthrough, all of the infective SARS-CoV-2 strains were found to be the BA.2 sub-lineage of omicron variant. This indicated that BA.2 variants were the predominant lineage circulating in the state during that wave. Moreover, the 13 individuals who were reinfected after booster had an IgG titer of 1,954.5 AU/ml and 45.2% of inhibitory neutralizing antibodies at BD0. This finding established that the presence of neutralizing antibodies does not guarantee protection against the omicron variant. Few earlier studies also found these escapes of SARS-CoV-2 omicron variants to antibody neutralization conferred by the currently available other vaccines (Cao et al., 2022; Kuhlmann et al., 2022; Planas et al., 2022; Yadav et al., 2022).

This study had a few limitations. First, all the participants were of a single nationality and same ethnic group, which restricted the generalization of the data. Second, the imbalance in the different age groups might lead to a discrepancy in the statistical significance. The median age of the cohort was 39 years; thus, the overall outcome might not be generalizable to children or older age groups. Finally, we lost a few participants permanently during this year-long follow-up. Many HCWs might have missed a specific timepoint and came back to give samples at the very next timepoint, but we could not include them in the final analysis.

To the best of our knowledge, this study was the first to prospectively and comparatively analyze the dynamicity and persistence of vaccine-induced antibodies over a period of 1 year among the Covaxin and Covishield recipients. We also considered age, sex, blood groups, and comorbidities to evaluate the long-term efficacy of these two vaccines. The findings from this longitudinal cohort study can help to implement vaccination strategies, particularly the need for a booster dose of the present vaccines. It could also aid in speculation about the requirement for more potent vaccine options or vaccine mandates to minimize the vaccine escape. The use of mixed or heterologous vaccines as boosters can be studied to evaluate the correlates of protection and sustainability of longer antibody responses. An extended study would also be helpful to understand the kinetics of cellular immunity in booster recipients.

## Data availability statement

All the data presented in this study are included in the article/Supplementary material, further inquiries can be directed to the corresponding authors. Sequencing data presented in the study are deposited in the GISAID repository, accession numbers: EPI_ISL_12363379, EPI_ISL_12363383, EPI_ISL_12363394, EPI_ISL_12363395, EPI_ISL_12363399, EPI_ISL_12363385, and EPI_ISL_12363393.

## Ethics statement

The studies involving human participants were reviewed and approved by the Institutional Human Ethical Committee of ICMR-Regional Medical Research Center, Bhubaneswar (ICMR-RMRCB/IHEC-2020/036 dated 07/11/2020). The patients/participants provided their written informed consent to participate in this study.

## Author contributions

DB and SP conceptualized the study. HC, MP, AS, and SJ collected blood samples, information from participants, and written informed consents. DeP, SSa, IM, and AM performed the laboratory tests. IP and SSu performed NGS. DeP and GD were involved in the data analysis. DeP and DB drafted the original manuscript. IP, SK, and SP edited and reviewed the manuscript. SB, NM, DiP, SR, SM, and DB supervised the study. All the authors read and reviewed the manuscript and gave their final approval.
