# Supplementary material for: Dynamicity and persistence of severe acute respiratory syndrome coronavirus-2 antibody response after double dose and the third dose with BBV-152 and AZD1222 vaccines: A prospective, longitudinal cohort study
Source: Front Microbiol. 2022 Aug 9;13:942659. doi: 10.3389/fmicb.2022.942659 (PMC9396971; doi:10.3389/fmicb.2022.942659)
Supplement: Supplementary file 2 [file Table_2.DOCX]

**Table S1.** Genome coverage and run details of the sequenced samples.

| Sample ID | Read length range (minimum to maximum) | Total reads length | 20X genome coverage | Accession Number |
| --- | --- | --- | --- | --- |
| BC-36 | 106-2165 | 791638 | 12.13 | NA |
| BC-39 | 108-8600 | 31104988 | 70.31 | NA |
| BC-40 | 104-1974 | 9449882 | 62.04 | NA |
| BC-62 | 124-5718 | 12533437 | 72.62 | EPI_ISL_12363383 |
| BC-65 | 125-2776 | 13773774 | 75.57 | EPI_ISL_12363385 |
| BC-71 | 154-8085 | 12832643 | 69.28 | EPI_ISL_12363393 |
| BC-74 | 147-10209 | 877096 | 37.04 | EPI_ISL_12363379 |
| BC-78 | 123-3550 | 6128767 | 65.09 | EPI_ISL_12363394 |
| BC-79 | 98-2838 | 5244728 | 66.05 | EPI_ISL_12363395 |
| BC-82 | 110-11929 | 2971112 | 57.0 | EPI_ISL_12363399 |
